# Serum-Based KRAS^G12/G13^ Mutation Detection Using Droplet Digital PCR: Clinical Implications and Limitations in Colorectal Adenocarcinoma With Tumor Heterogeneity

**DOI:** 10.3389/fonc.2020.604772

**Published:** 2021-01-11

**Authors:** Ju Seok Kim, Go Eun Bae, Seok-Hwan Kim, Min Kyung Choi, Min-Kyung Yeo

**Affiliations:** ^1^ Department of Internal Medicine, Chungnam National University School of Medicine, Daejeon, South Korea; ^2^ Department of Pathology, Chungnam National University School of Medicine, Daejeon, South Korea; ^3^ Department of Surgery, Chungnam National University School of Medicine, Daejeon, South Korea

**Keywords:** colorectal adenocarcinoma, cell free DNA, serum, KRAS, heterogeneity

## Abstract

**Background:**

Cell-free DNA (cfDNA) has arisen as an alternative target for evaluating somatic mutations in cancer. KRAS mutation status is critical for targeted therapy in colorectal adenocarcinoma (CRAC). We evaluated KRAS^G12/G13^ mutations in cfDNA extracted from serum and compared the results with KRAS^G12/G13^ mutations detected in tissue samples. We assessed the clinical significance of KRAS^G12/G13^ mutation in serum in regard to recurrence and metastasis of CRAC.

**Methods:**

A total of 146 CRAC patients were enrolled, and KRAS^G12/G13^ mutations were evaluated in 146 pairs of serum and tissue samples. In addition, 35 pairs of primary and metastatic CRAC tissue samples were evaluated for KRAS^G12/G13^ mutational status.

**Results:**

Detection of KRAS^G12/13^ mutation from serum and tissue had a 55% concordance rate, and serum detection had a sensitivity of 39.8%. Detection of the KRAS^G12/13^ mutation yielded a 14% discordance rate between primary and metastatic tissue. CRAC patients with mutant KRAS^G12/13^ mutation in serum but wild-type KRAS^G12/13^ in tissue had concurrent KRAS^G12/13^-mutant metastatic tumors, indicating spatial genetic heterogeneity. Changes in serum KRAS^G12/G13^ mutation status during postoperative follow-up were associated with recurrence. Conclusion: Although serum detection of the KRAS^G12/13^ mutation cannot substitute for detection in tissue, serum testing can support the interpretation of a CRAC patient’s status in regard to concurrent metastasis. Dynamic changes in serum KRAS^G12/13^ mutation status during follow-up indicated that cfDNA from serum represents a potential source for monitoring recurrence in CRAC patients.

## Introduction

Identification of genetic mutations in solid cancer is important for targeted therapy. For genotyping, a certain amount of tumor tissue acquired by biopsy or surgical resection is required. However, biopsy or excision of tumors can be difficult in some patients due to an unreachable tumor location, the risk of tumor spread, or potential clinical complications. Consequently, a more effective and non-invasive means of detecting genetic mutations is needed. To address this issue, cell-free DNA (cfDNA) has arisen as an alternative target for evaluating somatic mutations in cancer. Detection of mutations using cfDNA extracted from liquid samples, such as blood, urine, and saliva, is easily repeated and much less invasive than biopsy. In addition, the mutational status of cfDNA can be used to assess a cancer patient’s current status (1). Genetic mutation analysis using liquid samples has advanced rapidly in accordance with recently developed sensitive sequencing techniques (2). One such sequencing technique is droplet digital PCR (ddPCR), which is capable of sensitive detection of target DNA and quantification of mutations in small amounts of target DNA (3). ddPCR can be used to evaluate somatic mutations in liquid samples, including blood (4).

In colorectal cancer (CRC), KRAS mutational status is critical for targeted therapy (5) because it can predict the therapeutic response to anti-epidermal growth factor receptor (EGFR) treatment; consequently, KRAS genotyping is routine in patients with metastatic CRC. Tissue samples are commonly used for genotyping in CRC patients, but several studies have tried to establish the presence of KRAS mutations in cfDNA (6–8). Evaluation of mutation status using cfDNA is used primarily for genotyping; in addition, cfDNA has the advantage that it reflects tumor dynamics more closely than tissue samples. On the other hand, cfDNA also has drawbacks, including the fact that it is easily degraded and cannot be detected at low levels in samples (4). Clinical meaning of serum KRAS mutation has shown to have its own clinical implication apart from tissue KRAS related to prognosis (7).

Detection of KRAS mutations in cfDNA from CRC patient serum has been proposed, but the clinical implications and limitations of serum detection of KRAS mutations have not yet been clarified. This study sought to evaluate i) the concordance of detection of KRAS mutation between serum and tissue: can serum substitute for tissue in evaluation of KRAS mutation in CRC patients? ii) clinical implications of KRAS mutation status in serum: does the presence of KRAS mutation in serum, or the KRAS mutation fraction, have clinical implications in CRC patients? and iii) the change in KRAS status during follow-up: does KRAS status predict patient metastasis or recurrence in CRC? We evaluated KRAS mutation by ddPCR using serum and tissue samples from CRC patients and assessed the clinical significance of serum detection of the KRAS^G12/G13^ mutation.

## Materials and Methods

### KRAS^G12/G13^ Mutation Detection in Colorectal Adenocarcinoma Patients

This retrospective study included 146 colorectal adenocarcinoma (CRAC) patients who underwent surgical resection of primary colorectal tumors. Patients were diagnosed at the Chungnam National University Hospital (Daejeon, Korea) between January 2014 and December 2017; mean follow-up was 54 months. Pre-operative blood samples (within 1 week prior to the operation) from all patients were collected at the time of the first surgery, and 146 pairs of primary tumor samples were obtained from formalin-fixed, paraffin-embedded (FFPE) tissue blocks. Thirty-nine of the 146 patients exhibited concurrent liver metastases at the time of the first surgery; liver tissue samples were obtained by surgical tumorectomy from seven of the 39. Forty-seven patients had CRAC recurrence during follow-up, and follow-up blood samples were collected from 12 of the 47 patients (within a week prior to the second recurrent tumor operation) along with paired recurrent tumor samples. Clinical data of CRAC patients were available from the archives of the same institution. At the time of collection of pre-operative serum samples, no patients had received pre-operative chemo- or radiotherapy. Patients with stage III or higher CRAC who underwent curative resections received adjuvant FOLFOX (5-fluorouracil (FU) + oxaliplatin + leucovorin) with cetuximab (anti- epidermal growth factor receptor monoclonal antibody for tissue KRAS wild type) chemotherapy. An additional 35 CRAC patients with distant metastasis were evaluated for KRAS^G12/G13^ mutation. Thirty-five primary and metastatic tumor FFPE tissue samples were used to evaluate KRAS^G12/G13^ mutational status.

Serum and tumor tissue samples of CRAC patients were provided by the Biobank of Chungnam National University Hospital, a member of the Korea Biobank Network. This study was approved by the institutional review board of Chungnam National University Hospital (IRB file no. 2018-10-012-001). Because the study was retrospective, a waiver of consent was approved by the IRB.

### Serum KRAS^G12/G13^ Mutation Detection Using Droplet Digital Polymerase Chain Reaction

**Figure 1:**
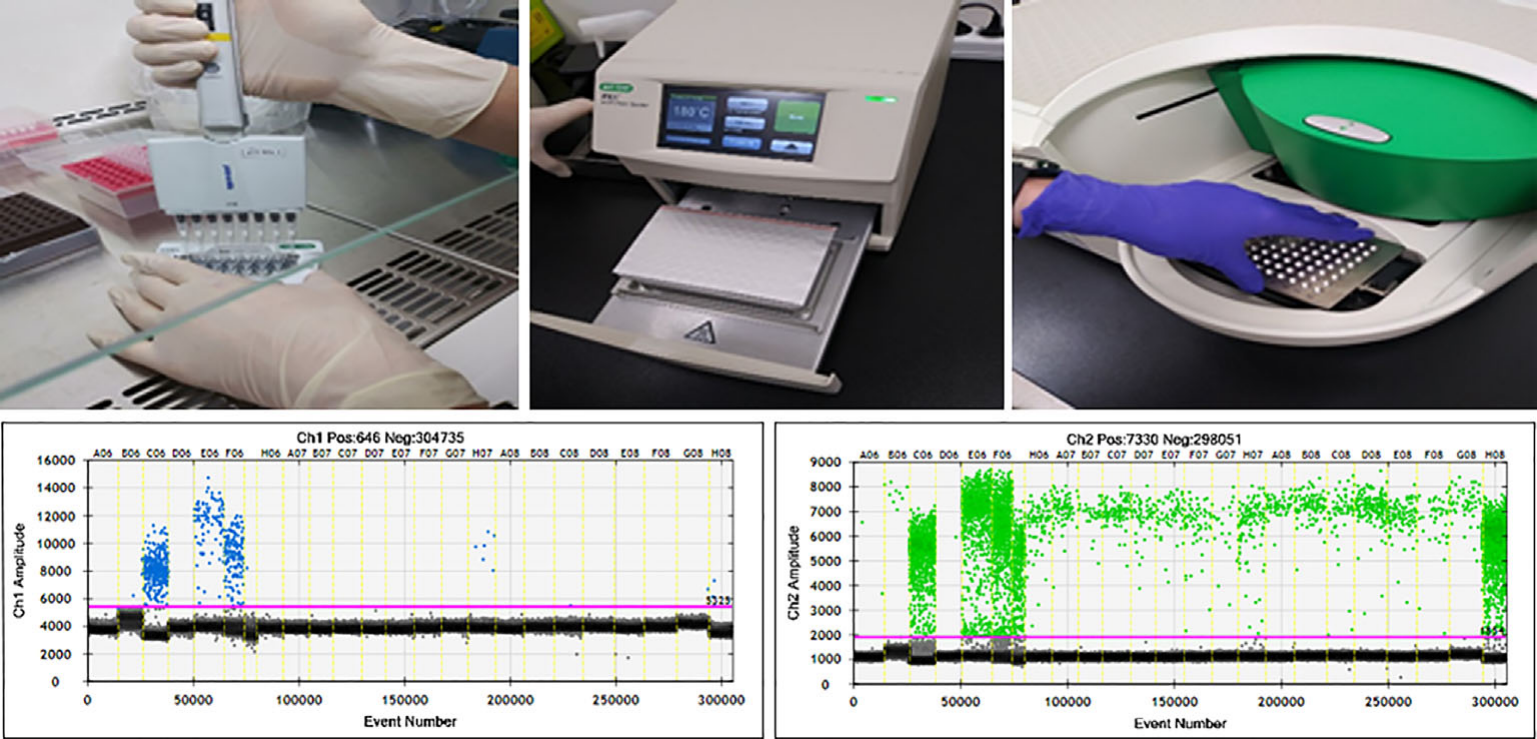
ddPCR workflow and results of ddPCR for detection of KRAS^G12/13^. Channel 1: fluorescence measurement of mutant probe labeled with 6‐fluorescein amidite (FAM) (lower left). Channel 2; wild-type probe labeled with hexachlorofluorescein (HEX) (lower right).

A total of 146 peripheral blood samples were collected, centrifuged to isolate serum, and stored in liquid nitrogen. cfDNA was extracted from 200 µl of stored serum using the QIAamp Circulating Nucleic Acid kit (Qiagen). Extracted cfDNA was eluted in 100 µl of Tris-EDTA buffer and diluted to 10 ng/µl. Sixteen serum samples from healthy people were used as negative controls. DNA extracted from CRAC serum was tested by ddPCR (QX200; Bio-Rad, Hercules, CA, USA) using the ddPCR Bio-Rad KRAS G12/G13 multiplex kit (#1863506). Reaction mixtures (ﬁnal volume, 20 µl) consisted of extracted DNA (1 μl), 2× SuperMix for probe (10 μl), KRAS screening probe (1 μl), and distilled water (8 µl). The mixture was loaded into a disposable droplet generator cartridge (Bio-Rad), and 70 µl of droplet generation oil for primers (Bio-Rad) was loaded into each of the eight oil wells (**Figure 1**). The cartridge was then placed inside the QX200 droplet generator (Bio-Rad), which partitioned each tissue sample into ~22,000 droplets per tissue sample. When droplet generation was complete, the droplets were transferred to a 96-well PCR plate. The plate was heat-sealed with foil and placed in a conventional thermal cycler (T100, Bio-Rad) using the following reaction conditions: 95°C for 10 min (1 cycle); 94°C for 30 s and 55°C for 1 min (40 cycles); 98°C for 10 min (1 cycle); and 4°C (hold). Cycled droplets were read individually on a QX200 droplet-reader (Bio-Rad). Samples were transferred to the QX200 for ﬂuorescence measurement of a mutant probe labeled with 6-ﬂuorescein amidite (FAM) and wild-type probe labeled with hexachloroﬂuorescein (HEX) (**Figure 1**). DNA from SW480 cells, which harbor the KRAS G12V mutation, served as a positive control; DNA from the leukocytes of heathy persons, DNA from HEK cells, and distilled water were used as negative controls.

The ddPCR platform used the QuantaSoft software (version 1.7; Bio-Rad) to calculate the number of positive and negative ﬂuorescence signals in droplets. Mutant allele frequency (MAF) was measured as the percentage of mutant droplets relative to the total (mutant + wild type). Samples from healthy volunteers contained no KRAS^G12/G13^ mutant droplets.

### Detection of KRAS^G12/G13^ in Tissue by Sanger Sequencing

A total of 146 primary tumor tissue slides were reviewed by two pathologists (M-KY and GEB), and representative tissue FFPE blocks were selected. FFPE tissue samples with minimum 1.0 x 1.0 x 0.3 cm tumor size at least 70% of tumor cell content were macro-dissected and sectioned. Four sections (5-mm thickness) of each qualifying tumor tissue sample were used for DNA extraction. Tumor tissue DNA was isolated from FFPE slides using he QIAamp DNA FFPE Tissue Kit (QIAGEN Korea, Seoul, Korea). Extracted DNA (20 ng) from CRAC tissue samples were subjected to Sanger sequencing (performed by Macrogen, Seoul, Korea). For detection of mutations in codons 12 and 13 of the *KRAS* gene, primer sequences were as follows: exon 2, 50-GTAAAACGACGGCCAGTGTGTGACATGTTCTAATATAGTCA-30 (forward) and 50-GCGGATAA CAATTTCACACAGGGAATGGTCCTGCACCAGTAA-30 (reverse); exon 3, 50-TAATACGACTCACTATAGGGGTGCTTAGTGGCCATTTGTC-30 (forward) and 50-GCTAGTTATTGC TCAGCGGTATGCATGGCATTAGCAAAG-30 (reverse). PCR ampliﬁcation conditions were as follows: 95°C for 5 min (1 cycle); 95°C for 30 s, 60°C for 30 s, and 72°C for 1 min (35 cycles); and 72°C for 7 min. PCR products were puriﬁed using MultiScreen-PCR_96_ filter plates (Millipore SAS, Molsheim, France). The puriﬁed PCR products were then Sanger sequenced on a 3730xl automated sequencer (Applied Biosystems, Foster City, CA, USA) using the BigDye terminator v3.1 sequencing kit. Nucleotide sequence data were analyzed using the Variant reporter software version 1.1 (Applied Biosystems).

An additional 35 CRAC patients with distant metastasis were evaluated for the KRAS^G12/G13^ mutation by Sanger sequencing. Thirty-five pairs of primary and metastatic tumor FFPE tissue samples were used to evaluate KRAS^G12/G13^ mutation status. Concordance and discordance in KRAS^G12/G13^ mutation status between primary and metastatic tumors were evaluated.

### Statistical Analysis

Detection of the KRAS^G12/G13^ mutation was compared between serum and tissue, and correlation was assessed by κ statistics (0.00–0.19, slight; 0.21–0.39, fair; 0.40–0.59; moderate agreement). Diagnostic value of the serum KRAS^G12/G13^ mutation (sensitivity, specificity, positive predictive value, and negative predictive value) were calculated for the detection of tissue KRAS^G12/G13^ mutation. The associations of serum KRAS^G12/G13^ detection with clinicopathological variables were examined using Spearman’s rank correlation, Mann-Whitney U-test, and Fisher’s exact test. All statistical analyses were performed using SPSS version 26.0 for Windows (SPSS Inc., Chicago, IL, USA) and MedCalc version 19.2.0 for Windows (MedCalc Software Ltd, Belgium).

## Results

### Comparison of KRAS^G12/G13^ Mutation Detection in Serum and Tissue

Paired pre-operative serum and tissue samples from a total of 146 CRAC patients were evaluated for detection of KRAS^G12/G13^ mutation (**Table 1**). We detected KRAS^G12/G13^ mutations in 46 of 146 (32%) serum samples and 98 of 146 (67%) tissue samples. Serum KRAS^G12/G13^ mutation were matched with tissue KRAS^G12/G13^ mutation in 39/98 (40%) of CRAC patients. Sixty six patients yielded discrepant KRAS^G12/G13^ status, with mutant KRAS^G12/G13^ in serum and wild-type KRAS^G12/G13^ in tissue; 59 patients had wild-type KRAS^G12/G13^ in serum and mutant KRAS^12/G13^ in tissue; and 7 patients (* in **Table 1**) had serum KRAS^G12/G13^ mutation without KRAS^G12/G13^ mutation in tissue. The κ agreement of serum and tissue KRAS^G12/G13^ detection was 0.198 (p = 0.002) and the concordance rate was 55%.

**Table 1:** Comparison of pre-operative serum and tissue KRAS^G12/13^ detection in CRAC patients (n = 146).

Tissue KRAS^G12/13^	Pre-operative serum KRAS^G12/13^ ddPCR	
Sanger sequencing	Mutant	Wild-type
Mutant	39 (85%)	59 (59%)
Wild	*7 (15%)	41 (41%)
Total	46 (32%)	100 (69%)

*Cases yielded discrepant KRAS^G12/G13^ status: mutant KRAS in serum and wild-type KRAS in tissue.

Next, we calculated the diagnostic value of serum KRAS^G12/G13^ mutation (detected by ddPCR) for prediction of tissue KRAS^G12/13^ mutation in the same patient (**Supplementary Table 1**). The sensitivity and specificity of the serum KRAS^G12/G13^ mutation were 39.8 and 85.44% for the detection of tissue KRAS^G12/G13^ mutation. The positive and negative predictive values of serum KRAS^G12/13^ mutation detection using ddPCR were 84.8 and 41.0%, respectively (**Supplementary Table 1**).

### Clinical Significance of Serum KRAS^G12/G13^ Mutation Detection

The clinical significance of serum KRAS^G12/G13^ detection was evaluated separately in CRAC patients with wild-type and mutant KRAS^G12/G13^ in tissue. The clinical significance of serum KRAS^G12/G13^ detection was then evaluated in CRAC patients with wild-type KRAS^G12/13^ in tissue (n = 48); clinico-pathological parameters are shown in **Table 2**. Detection of serum KRAS^G12/13^ was significantly related to concurrent metastasis (M1) (p = 0.004); seven patients with mutant KRAS^G12/13^ in serum but wild-type KRAS^G12/13^ in tissue had a distant metastasis at the time of primary colon cancer surgery. Serum KRAS^G12/G13^ detection was not correlated with tumor size, stage (T, N), or differentiation (p = 0.963, p = 0.329, p = 0.813, and p = 538, respectively).

**Table 2:** Clinical significance of preoperative Serum KRAS^G12/13^ status detected by ddPCR in CRAC patients with wild-type KRAS^G12/13^ in tissue (n = 48).

Characteristics inpatients with wild-type KRAS in tissue	Patients	Serum KRAS^G12/13^ status of patients with wild-type KRAS^G12/13^ in tissue		
	No. (%)	Mutant	Wild-type	P
Age (mean)	62 (100)	62 (15)	62 (85)	0.263
Sex				0.295
Male	33 (69)	6 (86)	27 (66)	
Female	15 (31)	1 (14)	14 (34)	
Size (cm)	5.0 (100)	5.3 (15)	5.0 (85)	0.963
T stage				0.329
T1+T2	5 (10)	0 (0)	5 (12)	
T3+T4	43 (90)	7 (100)	36 (88)	
N stage				0.813
N0	12 (25)	2 (29)	10 (24)	
N1+N2	36 (75)	5 (71)	31 (76)	
M stage				0.004
M0	24 (50)	0 (0)	24 (59)	
M1	24 (50)	*7 (100)	17 (42)	
Differentiation				0.538
WD+MD	44 (92)	6 (86)	38 (93)	
PD	4 (8)	1 (14)	3 (7)	
Postop Recurrence				0.597
Absent	23 (48)	4 (57)	19 (46)	
Present	25 (52)	3 (43)	22 (54)	

Postop, Post-operative; WD, well differentiated; MD, moderately differentiated; PD, poorly differentiated.

*7 cases described in **Table 3**.

The clinical significance of serum KRAS^G12/G13^ detection was evaluated in patients with mutant KRAS^G12/13^ in tissue (n = 98); clinico-pathological parameters are shown in **Supplementary Table 2**. Serum KRAS^G12/G13^ detection was not correlated with tumor size, stage (T, N, M), or differentiation (p = 0.334, p = 0.451, p = 1.000, p = 0.07, and p = 1.000, respectively). Serum KRAS^G12/G13^ MAF was not related to clinico-pathological parameters.

### Cases With Discordant KRASG12/13 Results: Mutant in Preoperative Serum and Wild Type in Primary Tissue

Seven CRAC patients had mutant KRAS^G12/13^ in serum but wild-type KRAS^G12/13^ in primary tissue. All seven had simultaneous liver metastases (**Table 3**). Five of the seven had KRAS^G12/13^ mutation in the metastatic tumor (liver) without mutation in primary tissue. The MAF of serum KRAS ranged from 0.53% to 10%.

**Table 3:** Seven discordant KRAS^G12/13^ (*Table 1) cases: pre-operative serum, matched primary and concurrent metastatic tissue.

Patient	Pre-operative serum KRAS MAF	Primary tumor (colon) KRAS Sanger sequencing	Concurrent tumor (liver) KRAS Sanger sequencing
D1	10	Wild-type	Codon 12
D2	4.88	Wild-type	Codon 12
D3	3.85	Wild-type	Codon 12
D4	2.53	Wild-type	Wild-type
D5	1.23	Wild-type	Codon 12/13
D6	1.19	Wild-type	Wild-type
D7	0.53	Wild-type	Wild-type

*MAF, mutant allele frequency.

### Dynamics of KRAS^G12/13^ Detection in Serum and in Matched Primary and Recurrent Tissue

**Figure 2:**
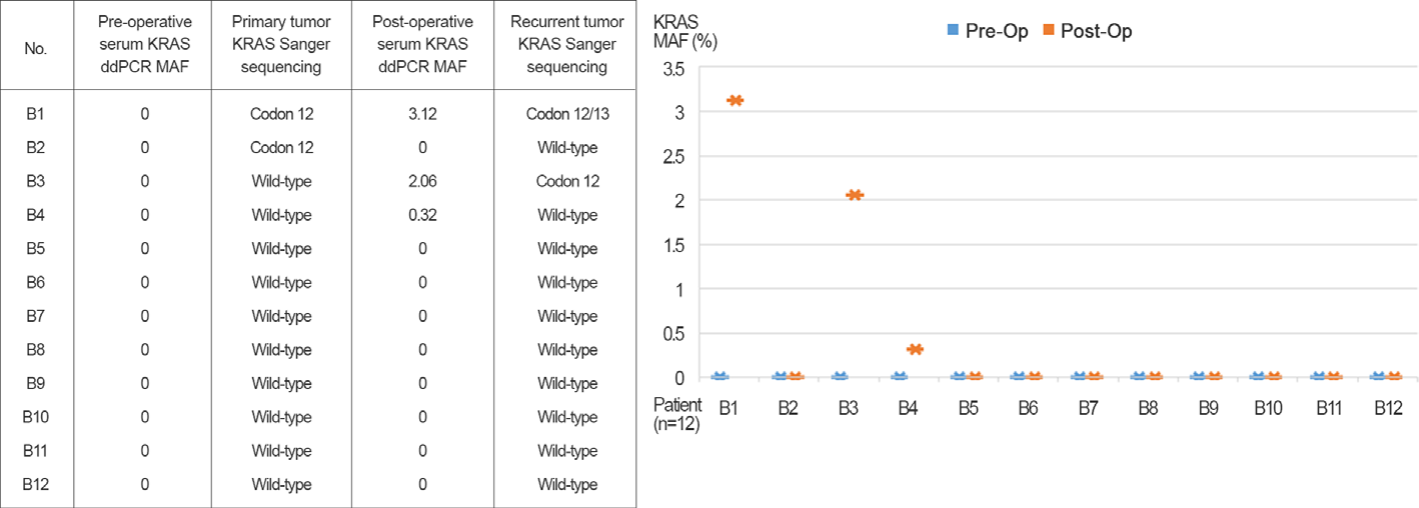
Pre- and post-operative serum KRAS^G12/13^ detected by ddPCR and primary and recurrent (as distant metastasis) tissue.

Pre- and post-operative serum samples from 12 patients were evaluated for KRAS^G12/13^ by ddPCR. Detection of tissue KRAS^G12/13^ status in matched primary (colon and rectum) and recurrent (as distant metastasis) tissue was assessed as shown in **Figure 2**. All patients received postoperative adjuvant chemotherapy, and patients with wild-type KRAS^G12/13^ (B3–B12) received additional cetuximab treatment. Patients B1 and B2 had a preoperative serum KRAS^G12/13^ MAF of 0 but were positive for tissue KRAS^G12/13^ mutation. In the follow-up period, all 12 patients had recurrence and underwent radical or palliative resection of recurrent tumors with distant metastasis. Post-operative serum KRAS^G12/13^ status was altered in several patients: three (B1, B3, and B4) became positive for serum KRAS^G12/13^ mutation, and two of those three (B1 and B3) were positive for tissue KRAS^G12/13^ mutation. The MAF of serum KRAS exhibited a recurrent tumor tissue KRAS^G12/13^ mutation (B1, B3) showed 3.12 and 2.06% MAF. The MAF of serum KRAS showed recurrent tumor tissue KRAS^G12/13^ wild type (B4) showed 0.32% MAF.

### Discordance in Detection of KRAS^G12/13^ Mutation Between Primary and Metastatic Colorectal Adenocarcinoma Tissue Samples

An additional 35 CRAC patients with distant metastasis were evaluated for KRAS^G12/G13^ mutation by Sanger sequencing. Pairs of 35 FFPE tissue samples from primary and metastatic tumors were used to evaluate KRAS^G12/G13^ mutation status (**Supplementary Table 1**). Twelve of 35 (34%) were KRAS^G12/13^ mutant, and 18 of 35 (51%) were KRAS^G12/G13^ wild type in both primary and metastatic tumor tissue. Five (T13–T17, * in **Supplementary Table 3**) out of 35 (14%) patients exhibited discordant KRAS^G12/13^ mutation status between primary and metastatic tissue. Of the five discordant cases, four (T14–T17) acquired KRAS^G12/G13^ mutation in distant tumor tissue samples (liver, lung bone, and ovary), whereas the primary tissue was wild type. The other patient (T13) was KRAS^G12/G13^ mutant in primary colon tissue but lost the mutation in distant tumor tissue (liver).

## Discussion

DNA fragments released by tumor cells can be detected in blood. Blood can be obtained easily and repeatably in the clinic. Hence, mutational analysis of cfDNA from blood represents an excellent alternative to tumor tissue samples. In this study, we evaluated the possibility of detecting KRAS^G12/G13^ mutation in serum samples from CRAC patients. KRAS^G12/G13^ mutation could be detected in 40% of serum samples from CRAC patients with KRAS^G12/G13^ mutation in tissue. Concordance between serum and tissue was limited (55%). Serum KRAS^G12/13^ mutation could detect tissue KRAS^G12/13^ mutation with a sensitivity of 39.8%, which is quite low. Previous studies reported concordance rates of 24.3% (7) and 50% when using DNA from circulating tumor cell samples (9). The serum samples could be used for genotyping but due to the low concordance rate were not an adequate substitute for tissue samples.

Mutational assessment of cfDNA in blood has prognostic significance (10, 11). The KRAS^G12/G13^ mutational status of CRAC patients had a different clinical impact depending on whether the mutation was detected in tissue or serum. Simultaneous KRAS mutation in both tumor and serum is associated with worse prognosis than when the mutation is only detected in tissue (8, 11). In this study, neither the presence of KRAS^G12/G13^ mutation in serum nor the MAF of KRAS^G12/G13^ had prognostic implications. Notably, serum KRAS^G12/13^ mutation was detected in 15% in patients who had wild-type KRAS^G12/13^ in tissue, and these patients was significantly related to M1 stage (concurrent metastasis).

We observed that KRAS^G12/G13^ mutation status was heterogeneous in serum and tissue, and considered to be related to concurrent metastasis. Tumor heterogeneity can be detected between different tumor regions, e.g., between primary and metastatic tumors (spatial heterogeneity) and within the primary tumor (intratumoral heterogeneity) (12, 13). Genetic discordance existed between primary and metastatic tumor that previous studies reported discordance of KRAS mutation status between primary colon and liver were approximately 5% (14, 15). We observed 86% concordance in KRAS^G12/13^ mutation status between primary and metastatic (liver, lung, bone, etc.) Two possibility of genetic discordancy in serum and tissue in our study has to be considered that intra-tumoral heterogeneity came from the primary tumor (we evaluated the representative section of tumor tissue) or spatial genetic heterogeneity between primary and metastatic tumor existed. All patients with mutant KRAS^G12/13^ in serum and wild-type KRAS^G12/13^ in tissue exhibited concurrent metastasis; accordingly, the metastatic tumor could be considered to be the source of the KRAS^G12/G13^ mutation. Patients with discordance between serum and tissue should be carefully monitored that patients need to be evaluated unidentified or hidden concurrent metastasis.

Mutational assessment of cfDNA in blood has the potential to predict recurrence or patient metastasis. KRAS mutations is acquired after chemotherapy as a resistance mechanism (16, 17). In the pre- and postoperative serum monitoring performed in this study, three patients exhibited conversion of post-operative serum KRAS^G12/13^ mutation status from preoperative wild type to postoperative mutant. Two of the three also had tissue KRAS^G12/13^ mutations. Changes in serum KRAS^G12/G13^ mutation status during postoperative follow-ups were related to recurrence. Dynamic changes in serum KRAS^G12/13^ mutation status during follow-up indicated that cfDNA from serum represents a potential source for monitoring recurrence in CRAC patients.

The present study had several limitations. Due to the small number of patients, the results provide less definitive conclusions regarding the effectiveness of ddPCR-based detection of serum KRAS^G12/G13^ status in CRAC patients. In our retrospective study, frozen stored serum samples could affect mutational output due to archiving status and time interval. Moreover, because changes in KRAS^G12/G13^ mutation occurred in only 3 of 12 patients, the ability to predict recurrence was limited. Serum KRAS^G12/G13^ status can give additional supportive information for the interpretation of CRAC patient status but must be considered along with other clinical and radiologic findings.

We compared the performance of KRAS^G12/G13^ somatic alterations in cfDNA with that of tissue samples. The use of ddPCR enables tracking of the appearance and disappearance of somatic alterations in serum-derived cfDNA. cfDNA mutational analysis captures tumor molecular heterogeneity, providing different view of a patient’s disease status. Because of the lack of follow-up samples, we cannot say whether KRAS^G12/G13^ mutations in cfDNA can be detected before radiological relapse. Hence, further studies involving larger numbers of patients and a prospective design are required.

## Data Availability Statement

The raw data supporting the conclusions of this article will be made available by the authors, without undue reservation.

## Ethics Statement

The studies involving human participants were reviewed and approved by the institutional review board of Chungnam National University Hospital (IRB file no. 2018-10-012-001). Because the study was retrospective, a waiver of consent was approved by the IRB.

## Author Contributions

JK provided the resources and contributed to the data curation. GB conceptualized and wrote, reviewed, and edited the manuscript. S-HK conceptualized and validated the study. MC conducted the formal analysis. M-KY supervised, wrote the original draft, and wrote, reviewed, and edited the manuscript. All authors have read and agreed to the published version of the manuscript. All authors contributed to the article and approved the submitted version.

## Funding

This study was supported by the research fund of Chungnam National University, grants from the Basic Science Research Program through the National Research Foundation of Korea (NRF) funded by the Ministry of Education (2017R1D1A1B04031187), and the Bio and Medical Technology Development Program of the National Research Foundation (NRF) funded by the Korean government (MSIT) (2019M3E5D1A02068558).

## Conflict of Interest

The authors declare that the research was conducted in the absence of any commercial or financial relationships that could be construed as a potential conflict of interest.
