# Supplementary material for: Serum-Based KRASG12/G13 Mutation Detection Using Droplet Digital PCR: Clinical Implications and Limitations in Colorectal Adenocarcinoma With Tumor Heterogeneity
Source: Front Oncol. 2021 Jan 11;10:604772. doi: 10.3389/fonc.2020.604772 (PMC7873888; doi:10.3389/fonc.2020.604772)
Supplement: Supplementary file 1 [file Table_1.docx]

Supplementary table 1. Diagnostic value of ddPCR detection of serum KRAS^G12/13^ mutation in predicting tissue KRAS^G12/13^ mutation

|  | Sensitivity | Specificity | Positive predictive value | Negative predictive value |
| --- | --- | --- | --- | --- |
| Serum KRAS^G12/13^ | 39.8% (30.04-50.18) | 85.4% (72.24-93.93) | 84.8% (72.93-92.01) | 41.0% (36.29-45.88) |

| Characteristics | Patients | Serum KRAS^G12/13^ status of tissue KRAS^G12/13^ mutant patients | | |
| --- | --- | --- | --- | --- |
|  | No. (%) | Mutant | Wild | P |
| Age (mean) | 67 (100) | 67 (37) | 67 (63) | 0.932 |
| Sex |  |  |  | 0.675 |
| Male | 55 (56) | 19 (53) | 36 (58) |  |
| Female | 43 (44) | 17 (47) | 26 (42) |  |
| Size (cm) | 4.8 | 5.1 (37) | 4.6 (63) | 0.334 |
| T stage |  |  |  | 0.451 |
| T1+T2 | 21 (21) | 6 (17) | 15 (24) |  |
| T3+T4 | 77 (79) | 30 (83) | 47 (76) |  |
| N stage |  |  |  | 1.000 |
| N0 | 58 (59) | 21(58) | 37 (60) |  |
| N1+N2 | 40 (41) | 15 (42) | 25(40) |  |
| M stage |  |  |  | 0.407 |
| M0 | 83 (85) | 32 (89) | 51 (82) |  |
| M1 | 15 (15) | 4 (11) | 11 (18) |  |
| Differentiation |  |  |  | 1.000 |
| WD+MD | 89 (91) | 33 (92) | 56 (90) |  |
| PD | 9 (9) | 3 (8) | 6 (10) |  |
| Postop Recurrence |  |  |  | 0.587 |
| Absent | 76 (78) | 29 (81) | 47 (76) |  |
| Present | 22 (22) | 7 (19) | 15 (24) |  |

Supplementary table 2. Clinical significance of preoperative serum KRAS^G12/13^ status detected by ddPCR in CRAC patients with mutant KRAS^G12/13^ in tissue (n=98)

Postop, post-operative; WD, well differentiated; MD, moderately differentiated; PD, poorly differentiated

*Cases yielded discrepant KRAS^G12/G13^ status: mutant KRAS in serum and wild-type KRAS in tissue

Supplementary table 3. Detection of KRAS^G12/13^ mutation in primary and metastatic CRAC tissue samples

| Patients ID | Tissue | KRAS^G12/13^ Sanger sequencing |
| --- | --- | --- |
| T1 | Colon | Codon 12 |
|  | Liver | Codon 12 |
| T2 | Colon | Codon 12 |
|  | Liver | Codon 12 |
| T3 | Colon | Codon 12 |
|  | Lung | Codon 12 |
| T4 | Rectum | Codon 12 |
|  | Liver | Codon 12 |
| T5 | Colon | Codon 12 |
|  | Lung | Codon 12 |
| T6 | Rectum | Codon 13 |
|  | Lung | Codon 13 |
| T7 | Rectum | Codon 12 |
|  | Lung | Codon 12 |
| T8 | Rectum | Codon 12 |
|  | Liver | Codon 12 |
|  | Lung | Codon 12 |
| T9 | Rectum | Codon 12 |
|  | Liver | Codon 12 |
|  | Lung | Codon 12 |
| T10 | Colon | Codon 12 |
|  | Liver | Codon 12 |
|  | Adrenal gland | Codon 12 |
| T11 | Colon | Codon 12 |
|  | Ovary | Codon 12 |
| T12 | Colon | Codon 13 |
|  | Peritoneum | Codon 13 |
| *T13 | Colon | Codon 12 |
|  | Liver | Wild-type |
| *T14 | Rectum | Wild-type |
|  | Liver | Codon 12 |
| *T15 | Colon | Wild-type |
|  | Lung | Codon 12 |
| *T16 | Rectum | Wild-type |
|  | Bone | Codon 12 |
| *T17 | Colon | Wild-type |
|  | Lymph Node | Wild-type |
|  | Liver | Codon 13 |
|  | Ovary | Codon 13 |
| T18 | Colon | Wild-type |
|  | Liver | Wild-type |
| T19 | Colon | Wild-type |
|  | Liver | Wild-type |
| T20 | Colon | Wild-type |
|  | Liver | Wild-type |
| T21 | Colon | Wild-type |
|  | Liver | Wild-type |
| T22 | Colon | Wild-type |
|  | Liver | Wild-type |
| T23 | Colon | Wild-type |
|  | Liver | Wild-type |
| T24 | Rectum | Wild-type |
|  | Liver | Wild-type |
| T25 | Rectum | Wild-type |
|  | Liver | Wild-type |
| T26 | Rectum | Wild-type |
|  | Liver | Wild-type |
| T27 | Colon | Wild-type |
|  | Liver_1 | Wild-type |
|  | Liver_2 | Wild-type |
| T28 | Colon | Wild-type |
|  | Lung | Wild-type |
| T29 | Colon | Wild-type |
|  | Lung | Wild-type |
| T30 | Rectum | Wild-type |
|  | Lung | Wild-type |
| T31 | Rectum | Wild-type |
|  | Lung | Wild-type |
| T32 | Colon | Wild-type |
|  | Lung right | Wild-type |
|  | Lung left | Wild-type |
| T33 | Rectum | Wild-type |
|  | Lung | Wild-type |
|  | Liver_1 | Wild-type |
|  | Liver_2 | Wild-type |
| T34 | Rectum | Wild-type |
|  | Lung left | Wild-type |
|  | Lung right_1 | Wild-type |
|  | Lung right_2 | Wild-type |
| T35 | Colon | Wild-type |
|  | Ovary | Wild-type |

* Cases yielded discrepant results regarding KRAS^G12/G13^ mutation status.
